# Response Assessment of Generic Tyrosine Kinase Inhibitors in Newly Diagnosed Chronic Myeloid Leukemia in Chronic Phase: A Prospective Study from a Tertiary Care Centre in North Eastern India

**DOI:** 10.7759/cureus.98893

**Published:** 2025-12-10

**Authors:** Ananya Choudhuri, Jina Bhattacharyya, Smita Das, Damodar Das, Neeraj Dhameja

**Affiliations:** 1 Hematology, Anandoloke Multispeciality Hospital, Siliguri, IND; 2 Hematology, Gauhati Medical College and Hospital, Guwahati, IND; 3 Pathology, Institute of Medical Sciences, Banaras Hindu University, Varanasi, IND

**Keywords:** chronic myelogenous leukemia, chronic myeloid leukemia, generic tyrosine kinase inhibitors, hematopoietic stem cells, myeloproliferative neoplasm

## Abstract

This study evaluated the efficacy of generic tyrosine kinase inhibitors (TKIs) in newly diagnosed chronic myeloid leukemia in chronic phase (CML-CP) and analyzed Breakpoint Cluster Region-Abelson *(BCR-ABL)* kinase domain mutations in patients exhibiting TKI resistance. Eighty-five recently diagnosed CML patients (65 males and 20 female participants) of mean age 39.93±10.13 years, who had CP, were enrolled in this prospective study, and their response to generic TKI was evaluated. Furthermore, *BCR-ABL* TK domain mutations were assessed in individuals who did not respond well to generic TKI. Male preponderance was observed, representing 65 (76.5%) and 79 (92.9%) of the patients from Assam. The mean baseline hemoglobin (Hb) was 7.6 g/dl, and the mean blast percentage was 2.97. Following treatment with a generic TKI, mean *BCR‑ABL* levels fell by 10% at three months in 62 cases (77.5%), by 1% at six months in 64 cases (80%), and to 0.1% by 12 months in 48 cases (60%). A complete hematological response (CHR) was noted in 80 (94.11%) of the patients. The non-compliance rate to TKI in this study was seen in 10 (11.76%) out of the 85 cases. The most common drug-related hematological adverse effect was neutropenia in seven (8.2%) and anemia in five (5.9%) patients, respectively. The most common drug-related non-hematological adverse effect was edema in 12 (14.1%) and fatigue in 10 (11.7%) patients. Significant clinical, hematological, and molecular responses were reported by the study. The drug was found to be easily tolerated, and any negative effects observed were effectively managed with appropriate care.

## Introduction

Chronic myeloid leukemia (CML), also known as chronic myelogenous leukemia, is a disorder of hematopoietic stem cells (HSCs), which are involved in the production of blood cells. This disorder falls within the category of myeloproliferative neoplasm (MPN), which is characterized by the abnormal proliferation of blood cells, either red blood cells (RBCs), white blood cells (WBCs) or platelets. The t(9;22)(q34.q11.2) translocation forming the *BCR-ABL1* fusion gene is a hallmark of CML. The Breakpoint Cluster Region-Abelson *(BCR-ABL)* protein is a constitutively active tyrosine kinase that activates downstream pathways like Rat sarcoma (*RAS*), Janus kinase-signal transducer and activator of transcription 5 *(JAK-STAT5)*, Phosphoinositide 3-kinase-protein kinase B *(PIK3-AKT)*, leading to cell division and blocking apoptosis [1]. This fusion oncogene's main effect is the ongoing stimulation of the tyrosine kinase pathway, which causes the mutant HSCs to proliferate more than normal HSCs and gradually replace normal HSCs [2]. By inducing the tyrosine kinase enzyme, the *BCR-ABL* gene also contributes to genetic instability by causing hematopoietic cells, particularly those of the myeloid lineage present in the bone marrow stroma, to proliferate, change, suppress cell death, and disrupt cell adhesion. One important factor in determining the viability and proliferation of leukemic cells is the expression of the *BCR-ABL* gene [3].

The yearly incidence of CML in India ranges from 0.8 to 2.2 cases per 100,000 individuals [4]. The advent of tyrosine kinase inhibitors (TKIs) has changed the method of CML treatment dramatically. The life-expectancy of people suffering from CML is comparable to that of the populace at large. Furthermore, it is expected that 80% to 90% of these individuals will survive for 10 years [5]. Recently, several generic TKIs have been approved for use as the initial treatment for patients who have recently been diagnosed with chronic myeloid leukemia in chronic phase (CML-CP). This category includes first-generation TKIs such as imatinib, along with second-generation TKIs (SG-TKIs) like nilotinib, dasatinib, and bosutinib. SG-TKIs had markedly greater and more rapid molecular response compared to first-generation imatinib, without any disparity in long-term survival [6].

Imatinib is the first-line treatment for CML-CP, according to the groundbreaking International Randomized Study of Interferon and STI571 (IRIS). It revealed that after 12 months of treatment, 69% of patients receiving front-line imatinib had a complete cytogenetic response (CCyR), and 57% of them had a major molecular response (MMR). Some patients (7.9%), on the other hand, advanced to blastic crisis (BC) or accelerated phase (AP) [7]. TKI usage has significantly increased the survival rates of those with CML-CP. Approximately 20-30% of patients develop resistance to TKI therapy in due course of time, often due to *BCR-ABL1 *kinase domain, most commonly *T315I, F317L, E255K,* etc. [8]. Resistance despite the suppression of *BCR-ABL1* implies clonal evolution. Certain additional mutations can get activated, like the *BCR-ABL1* kinase domain mutation. In addition, 5-10% of patients stop treatment due to low tolerability [9].

With the availability of generic TKIs since 2003 and because of the marked reduction in the cost of treatment, they are now the choice of front-line therapy in CML. Although there have been several studies regarding the safety and efficacy of TKIs in the West, there has not been much data about the safety, efficacy, and toxicity profile of generic TKIs among the Indian population, particularly in northeastern India. Thus, the present study aimed to investigate the response of generic TKIs in patients with newly diagnosed CML-CP and to analyze *BCR-ABL* TK domain mutations in CML-CP patients with TKI failure.

## Materials and methods

This prospective, observational study was conducted from April 2023 to August 2024 among 85 patients in the Department of Clinical Hematology in a tertiary care medical institution in Eastern India. The Institutional Ethics Committee of Gauhati Medical College and Hospital, Guwahati, provided its approval (number: 190/2007/Pt-II/June-2023/2) before commencement of the study. Every participant enrolled in the study provided written informed consent. Inclusion criteria were newly diagnosed cases of CML patients in CP, and exclusion criteria consisted of CML cases in AP and BC, non-consenting patients, and pregnant patients.

Study methodology

Informed consent was obtained from every patient before examination and commencement of the study. A thorough record, including demographic data, which included age at presentation, sex, residence, and religion, was made. In addition to demographic profiles, clinical examination findings, including spleen size and laboratory parameters, comprising complete blood count (CBC) with differentials and blood biochemical profile, were recorded. All patients had bone marrow aspiration (BMA) and bone marrow biopsy (BMBx) at diagnosis, in addition to standard cytogenetics using karyotyping. Furthermore, to ascertain whether the *BCR-ABL1* transcript types (e13a2 or e14a2) were present in any patient, qualitative reverse transcriptase-polymerase chain reaction (RT-PCR) was carried out on the bone marrow or peripheral blood samples.

The European Treatment and Outcome Study (EUTOS) Long-Term Survival (ELTS) score was calculated using the online calculator [10]. Patients were prognosticated using the ELTS score according to their age, platelet count, spleen size, and blast percentage [11]. The patients were followed up at three, six, and 12 months after the initiation of generic TKIs. In case of no response to generic TKIs at the end of 12 months of treatment or progressive elevation in the percentage of *BCR-ABL1* (International Scale or IS%), patients were analyzed for *kinase domain (KD)* mutation by sequencing.

The response was assessed by measuring the *BCR-ABL* level as per European LeukemiaNet (ELN) CML response criteria [12] and the complete hematological response (CHR) was also assessed. A CHR was defined by a platelet count of less than 450,000/mm³, a WBC count of 10,000/mm³, absence of immature granulocytes or basophilia in the differential count, and a spleen that could not be palpated. Molecular response categories included a complete molecular response (CMR), which indicated no detectable *BCR-ABL* transcripts and a major molecular response (MMR), described as a drop in *BCR-ABL* transcript levels of more than three log (equivalent to a 1000-fold decrease) from the baseline standard established by the laboratory. *BCR-ABL* levels were expressed as a percentage ratio of the *BCR-ABL* transcript to the ABL1 transcript, reported on the IS. Patients who achieved CHR and remained clinically stable but did not accomplish either MMR or CMR after 12 months were considered resistant to treatment. Molecular relapse was defined as *BCR-ABL/ABL* levels detected with an elevation by twofold in three consecutive samples collected at least one month apart or a fivefold increase in two consecutive samples.

Detection of *BCR-ABL* was performed using real-time quantitative PCR (RQ-PCR) with the TRUPCR BCR-ABL kit (3B BlackBio Dx Limited, Bhopal, India), which is used to both identify and quantify *BCR-ABL* fusion transcripts in patients with CML. The principle of this method relies on the kit’s specificity for the *BCR-ABL* fusion gene resulting from the Philadelphia chromosome translocation (9;22) [13]. It utilizes quantitative RT-PCR to amplify *BCR-ABL* target sequences, with specific fluorescence reporters allowing for real-time quantification of the amplified product. An internal control, such as Abelson kinase (ABL) or Glyceraldehyde-3-phosphate dehydrogenase (GAPDH), is included in the kit to ensure PCR efficacy and to normalize for variations in sample processing.

For *BCR-ABL KD* mutation sequencing, Sanger sequencing was employed using template DNA, primers, DNA polymerase, deoxynucleoside triphosphate (dNTPs), and a small amount of fluorescently labeled dideoxynucleoside triphosphate (ddNTPs) in the reaction mixture [14]. Following standard steps, the reaction mix was first denatured to separate DNA strands and then cooled for primers to anneal to the single-stranded DNA. During the extension step, DNA polymerase added complementary dNTPs, with the occasional incorporation of a chain-terminating, labeled ddNTP. The resulting DNA fragments of varying lengths were separated by capillary electrophoresis, with each terminating at a labeled ddNTP that corresponded to a specific nucleotide. Fluorescent signals produced as DNA fragments passed through a detector were recorded, enabling determination of the DNA sequence according to the order of detected ddNTPs, and the output from the detection system was analyzed to determine the final DNA sequence.

Statistical analysis

Data were analyzed using IBM SPSS Statistics for Windows, Version 20 (Released 2011; IBM Corp., Armonk, New York, United States). Normality of data was checked using the Kolmogorov-Smirnov test and Shapiro-Wilk test. The response of parameters was analyzed through Friedman test, Mann-Whitney U test and unpaired Student t-test. Significance was considered for p<0.05.

## Results

The mean age of the patients was 39.93±10.13 years. The majority, 47 out of 85 patients (55.3%), were in the 20-40 years age group (Table 1).

**Table 1:** Age-wise distribution of the study participants

Age category (years)	Frequency	Percentage (%)
<20	1	1.2
20-40	47	55.3
>40	37	43.5
Total	85	100.0

Male preponderance was observed in the current study, with 65 male (76.5%) and 20 female (23.5%) participants. Majority of the patients (n=79; 92.9%) were from Assam (Table 2).

**Table 2:** Geographical location among the study population

Geographical location	Frequency	Percentage (%)
Arunachal Pradesh	1	1.2
Assam	79	92.9
Manipur	2	2.4
Mizoram	1	1.2
Nagaland	2	2.4

Incidental detection was noted in six patients (7.1%), easy fatigability in 53 patients (62.4%), swelling of the abdomen in 42 patients (49.41%), and abdominal pain in 10 patients (11.8%). Weight loss was observed in seven patients (8.2%) and excessive sweating in three patients (3.5%). Splenomegaly was present in all 85 (100%) cases. The mean size of spleen was 9.04 cm below the left costal margin (range 5-15 cm).

Baseline hematological variables of the patients are displayed in Table 3.

**Table 3:** Baseline hematological parameters among the patients IQR: interquartile range

Biochemical parameters	Median (IQR)
Hemoglobin (g/dL)	7.60 (7.2-8.2)
Total leucocyte count (X10^9^/L)	378000.00 (320350.00-435000.00)
Platelets (per µL)	500000.00 (448000.00-550000.00)
Basophils (%)	5.00 (4.00-6.00)
Blast (%)	3.00 (2.00-4.00)

The median hemoglobin (Hb) level among the patients was 7.6 g/dL (interquartile range or IQR, 7.2-8.2), which is indicative of anemia. The median of total leucocyte count (TLC) was 378000.00 X10^9^/L (IQR, 22.2-37.7), median of basophil and blast was 5.00% (IQR, 4.00-6.00) and 3.00% (IQR, 2.00-4.00) respectively.

Based on their ELTS scores, out of the 85 patients, 51 (60%) were stratified into intermediate risk, three (3.5%) into high risk, and 31 patients (36.4%) into low risk. The baseline karyotype was t(9;22) (q34q11.2), noted in all cases (100%). Additional high-risk cytogenetics was noted in two (2.35%) out of the 85 patients. The most common additional cytogenetic risk factor was 3q26.2. Treatment with imatinib was started in the low- and intermediate-risk patients in 82 (96.4%) out of 85 cases. Dasatinib was started in three high-risk cases (3.52%) out of the total cohort. CHR was achieved in 80 (94.11%) patients, which included 77 patients on imatinib and three patients on dasatinib.

**Figure 1:**
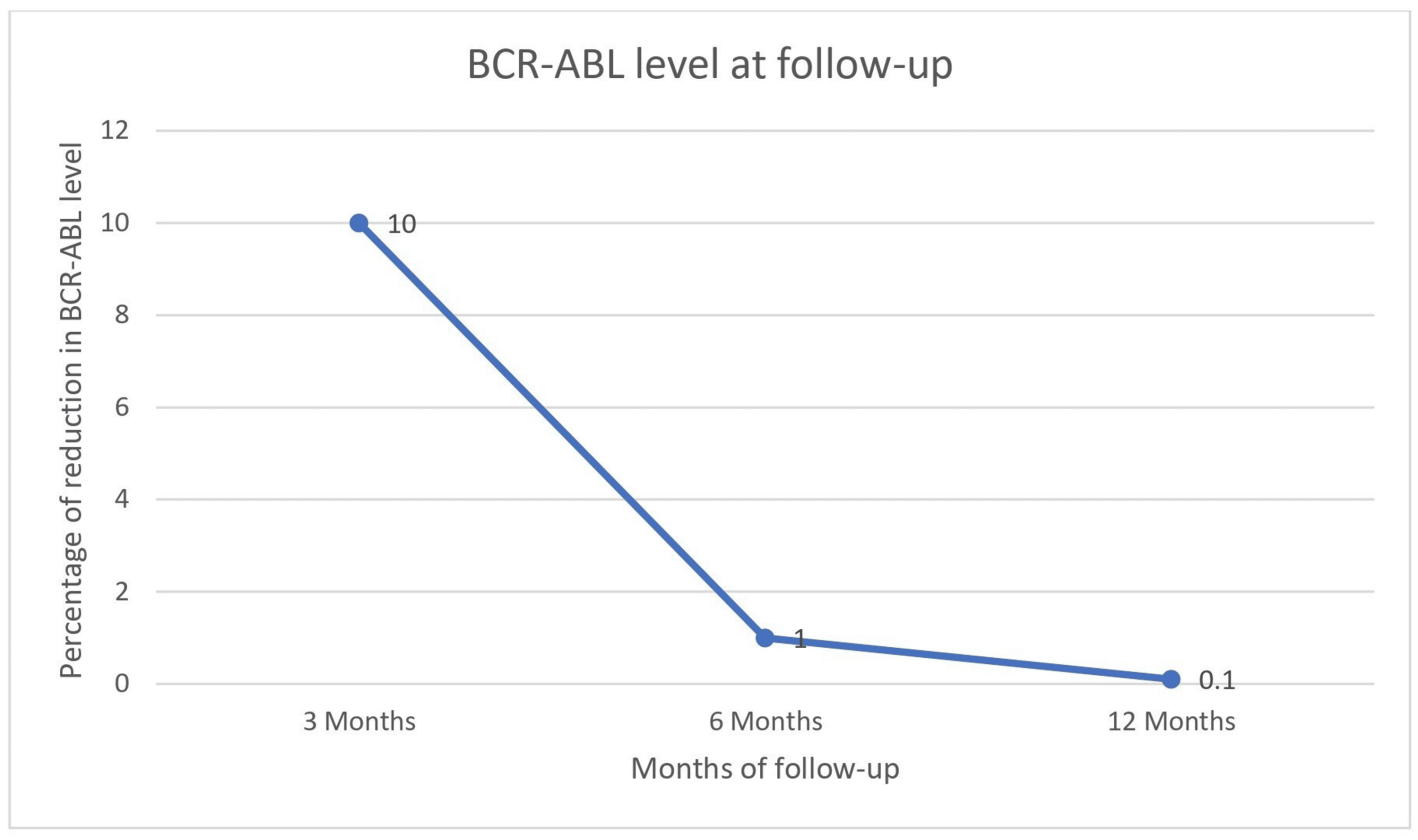
BCR-ABL levels during the follow-up BCR-ABL: Breakpoint Cluster Region-Abelson

Figure 1 depicts *BCR-ABL* levels during the follow-up.

The levels decreased to less than 10% at the three-month interval in 59 out of 77 imatinib cases and all the three dasatinib cases, which contributed to 62 (77.5%) out of the 80 cases. It reduced further to less than 1% at six months in 62 out of 77 imatinib cases and two out of the three dasatinib cases, which accounted for 64 (80%) of the 80 cases. At 12 months, it reduced to less than 0.1% in 46 out of 77 imatinib patients and two out of the three dasatinib patients, which contributed to 48 out of the 80 cases (60%), and was statistically significant ($\chi$^2^ (2) = 150.871, p=0.0001). CCyR was noted in 64 cases (80%). However, MMR was noted in 48 (60%) cases.

**Figure 2:**
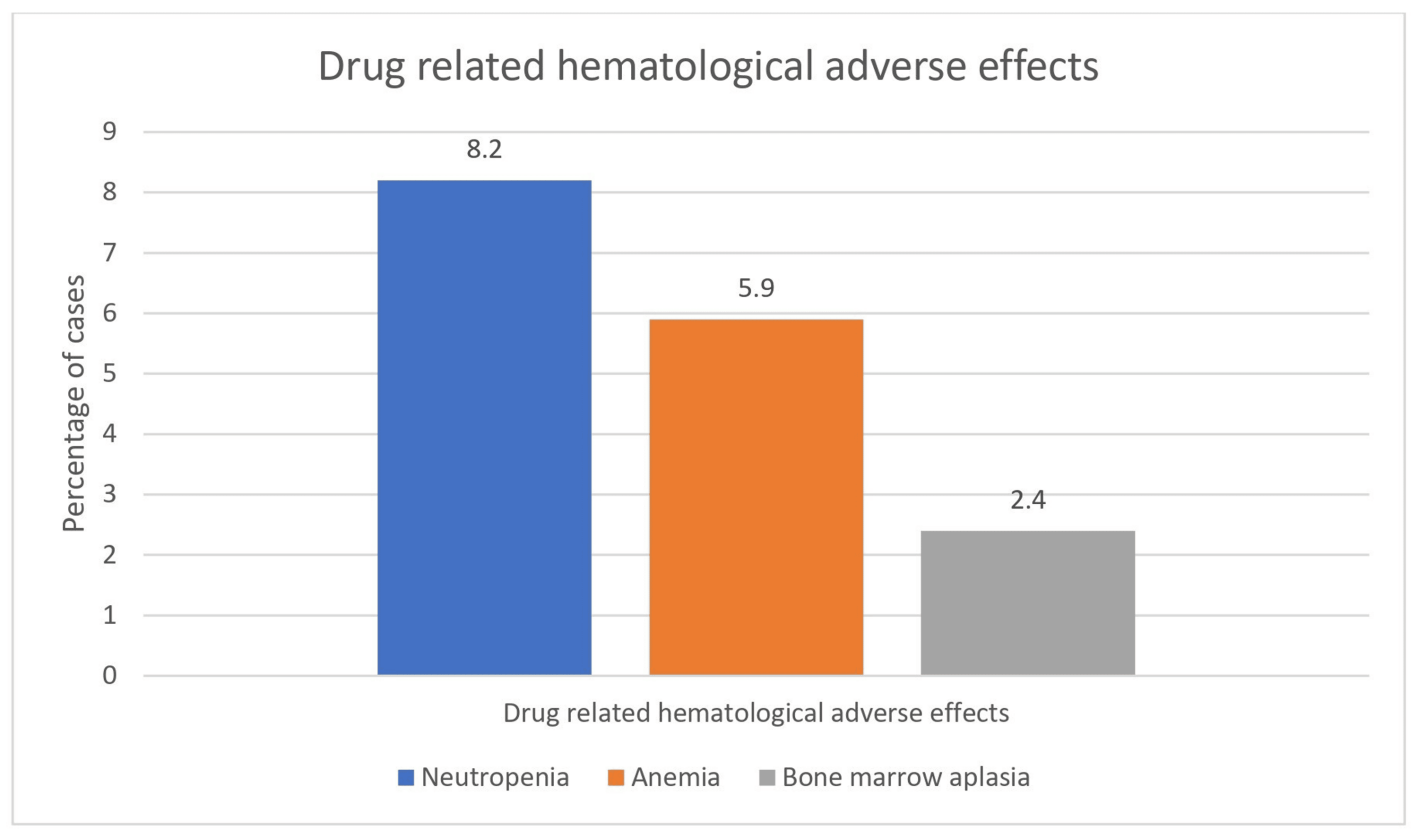
Drug-related hematological adverse effects

The most drug-related typical hematological adverse effect was neutropenia in seven (8.2%) cases, followed by anemia and bone marrow aplasia in five (5.9%) and two (2.4%) cases, respectively, depicted in Figure 2.

**Figure 3:**
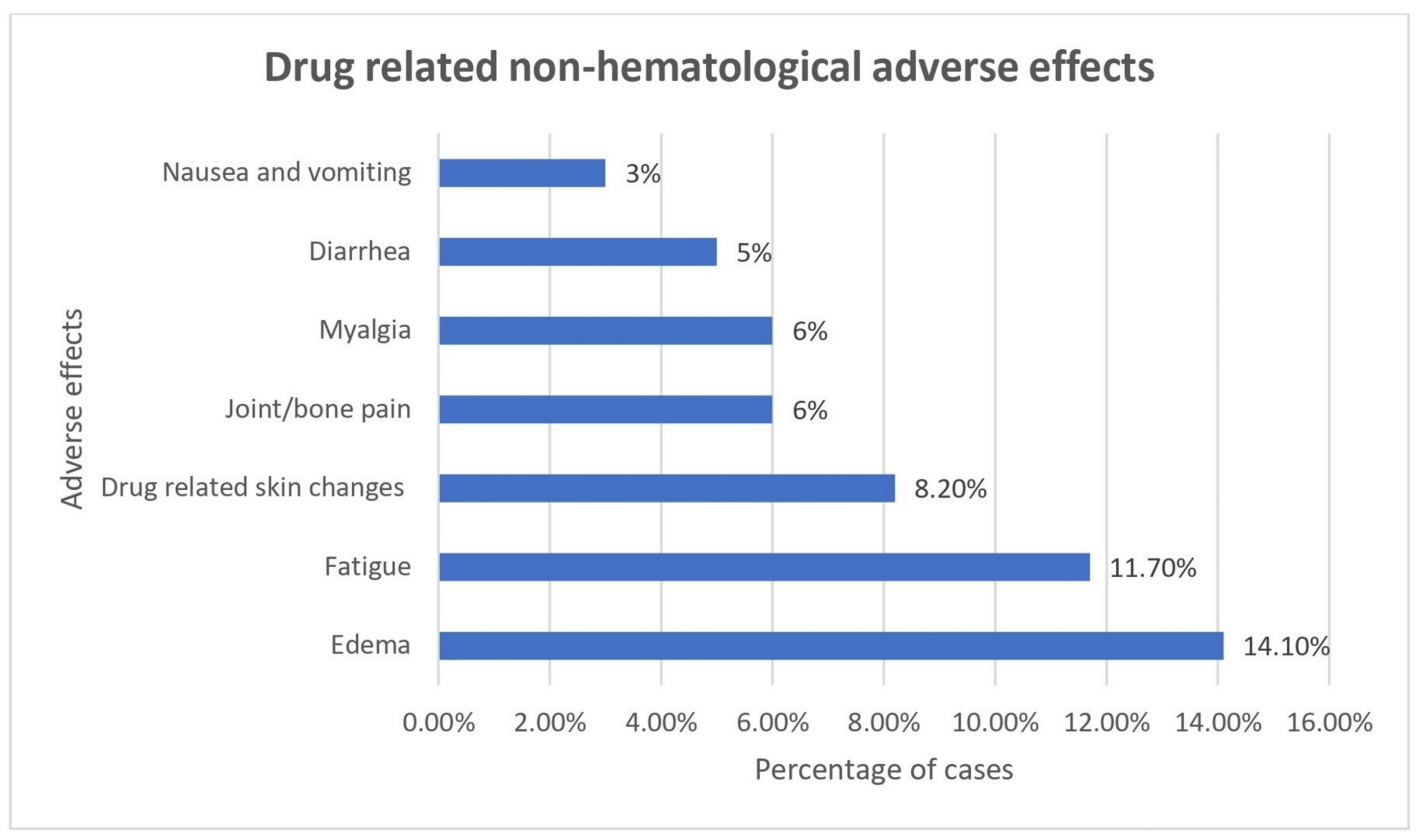
Drug-related non-hematological adverse effects

Out of seven cases of neutropenia, five had mild (grade 1-2) and two cases had severe (grade 3-4) neutropenia as per Common Terminology Criteria for Adverse Events (CTCAE) score [15]. Three out of the five cases of anemia were mild (grade 1-2), and two cases had severe (grade 3-4) anemia as per CTCAE score, requiring repeated blood transfusions. Bone marrow aplasia was noted in two cases, which was mild (grade 1-2) as per the CTCAE score. The most common drug-related non-hematological adverse effect was edema in 12 cases (14.1%), followed by fatigue in 10 cases (11.7%) and drug-related skin changes in seven (8.2%) cases (Figure 3).

All the adverse effects were mild (grade 1-2) as per the CTCAE score.

Prevalence of the *KD* mutation was discovered in this study; out of 32 cases who did not achieve MMR, 15 cases (42.85%) were positive for the *KD* mutation. The most common *KD* mutation was *F317L* in eight (53.33%) cases, followed by a combination of *c.C944T, T315I, F317L*, and *T315I* representing 13.33% of the cases. Out of 85 cases, 10 (11.76%) were non-compliant to treatment. In this study, all the noncompliant cases were males. Lack of understanding regarding the therapy and financial constraints had been among the most frequent causes of noncompliance. 

Table 4 illustrates a comparison of the biochemical indices between compliant and noncompliant patients with CML.

**Table 4:** Comparison of the biochemical indices between the compliant and non-compliant patients with chronic myeloid leukemia S: Significant (p<0.05); NS: Nonsignificant (p>0.05); SD: Standard deviation; Hb: Hemoglobin; TLC: total leukocyte count. The values of compliant and non-compliant patients with chronic myeloid leukemia are expressed as mean rank obtained from Mann-Whitney U test.

Variables	Compliant (n=79)		Non-compliant (n=6)		U value	p value
	Mean±SD (Range)	Mean Rank	Mean±SD (Range)	Mean Rank		
Spleen size (cm)	8.96±2.33 (8.00-10.00)	42.38	10.16±2.85 (8.00-12.75)	51.17	188.00	0.39^NS^
Hb (g/dL)	7.54±0.78 (7.20-8.20)	41.96	8.00±0.52 (7.50-8.45)	56.67	155.00	0.15^NS^
TLC (× 10^9^/L)	358259.01±94833.63 (320000.00-420000.00)	41.53	464666.66±109993.93 (342500.00-562500.00)	62.33	121.00	0.04^S^
Platelets (per µL)	486367.46±101296.16 (450000.00-550000.00)	43.61	451166.66±104468.97 (340000.00-545000.00)	34.92	188.50	0.40^NS^
Blast (%)	2.92±1.17 (2.00-4.00)	41.58	3.66±0.51 (3.00-4.00)	61.67	125.00	0.04^S^
Basophils (%)	4.83±0.92 (4.00-6.00)	43.31	4.66±0.81 (4.00-5.25)	38.92	212.50	0.65^NS^

There was no significant variation in the age (t=0.35, p=0.72) between the compliant (39.82±10.07 years) and non-compliant (41.33±11.77 years) patients. There was a statistically higher total leukocyte count (U=121.00, p=0.04) and blast% (U=125.00, p=0.04) in the non-compliant patients compared to the patients compliant to generic TKI treatment. The other biochemical indices were found to be statistically non-significant (p>0.05; Table 4).

## Discussion

The present study aimed to evaluate the therapeutic response of patients diagnosed with CML-CP when treated with generic TKIs. In addition, *BCR-ABL *tyrosine kinase domain mutations were assessed in patients who experienced TKI therapy failure. In terms of clinical presentation, the most common complaint noted among participants was fatigue, reported by 53 patients (62.4%), followed by abdominal lump in 42 (49.41%) patients and abdominal pain in 10 (11.8%) patients. These results are consistent with observations by Singh et al. [16] and Phukan et al. [17], where fatigue emerged as a predominant symptom in approximately 42.6% of the cases. On physical examination, splenomegaly was universally present in all 85 patients (100%), aligning with findings from Singh et al. [16] and Phukan et al. [17], who reported splenomegaly in 83.2% and 100% cases, respectively. This ongoing observation highlights splenomegaly as a defining clinical characteristic of CML-CP.

Hematological parameters at baseline in our study demonstrated a mean Hb level of 7.57 g/dl, a TLC of 3.71 X 10⁹/L, basophil percentage of 4.82%, and blast percentage of 2.97%. Comparatively, Singh et al. [16] observed Hb levels at 10.2±2 g/dL and TLC at 107,524.9 cells/mm³, while Phukan et al. [17] reported circulating blasts at 2%, correlating well with our findings. Cytogenetic analysis via karyotyping was conducted on all patients (100%). However, additional cytogenetic abnormalities were detected in only three (3.5%) cases, revealing a 3q26.2 abnormality indicative of poor prognosis. In contrast, trisomy 8 was found to be the most common cytogenetic abnormality by Kumar et al. [18]. Our findings potentially highlight regional variations in cytogenetic profiles within northeastern India, although the small sample size limits definitive conclusions.

The ELTS score was utilized for risk stratification among all participants [10]. Patients categorized as low or intermediate risk commenced imatinib therapy, whereas high-risk individuals received dasatinib, considering their younger age (<40 years), presence of adverse cytogenetics, absence of comorbidities, and preference for early deep molecular response. The use of the ELTS score, currently recommended by the ELN, offers improved predictive accuracy for survival outcomes and tailors treatment more effectively than older scoring systems like Sokal [11]. Dasatinib was started in 3.52% of the high-risk cases and imatinib in 96.4% of low- and intermediate-risk patients in our study. These tactics support the superiority of ELTS scoring over Sokal score and are consistent with protocols outlined by Eriko et al. [19] and Pfirrmann et al. [11]. Contrarily, studies by Singh et al. [16] and Phukan et al. [17] relied solely on generic imatinib without structured risk stratification.

Therapeutic responses were promising: 80 out of 85 patients (94.11%) achieved CHR. The remaining five patients (5.88%) likely failed due to poor compliance, limited understanding of the therapy, or logistical challenges. Chandey et al. [20] reported CHR in 88.8% at the three-month follow-up, comparable to our data. Nasser et al. [21] observed a lower CHR rate (68.5%) after one year, while Razmkhah et al. [22] reported a 90% CHR rate after eight months of treatment. The study by Nasser et al. [21] focused on patient adherence to imatinib treatment for CML and reported that almost half (43%) of patients exhibited low adherence.

Molecular response assessments revealed significant reductions in *BCR-ABL* transcript levels. At three months, 62 patients (77.5%) achieved ≤10%, 64 patients (80%) achieved ≤1% at six months, and 48 patients (60%) achieved ≤0.1% at 12 months. This progressive reduction was statistically significant (p=0.0001). By 12 months, 55.4% of patients had achieved MMR, according to Singh et al. [16]. Similarly, the IRIS trial [23] reported approximately 50.2% of patients achieving significant molecular remission after one year. Nair et al. [24] presented MMR rates of 6.3% and 25.8% at six and twelve months, respectively.

All patients in this study were administered generic imatinib provided via government supply. Following the patent expiration of original imatinib (Gleevec) in 2016, generic alternatives became accessible and affordable, enabling broader treatment availability. Notably, due to multiple manufacturers and frequent brand changes during the study period, detailed manufacturer data was not specified. Thus, the overall effectiveness and safety profile of generic imatinib were evaluated in this study. Eskazan et al. [25] highlighted that optimal responses at three and six months were comparable between the original and generic imatinib. Lejniece et al. [26] demonstrated sustained MMR over 24 months following a switch from original to generic imatinib. Danthala et al. [27] analyzed data from 1812 patients, confirming comparable efficacy and safety profiles between generic and original formulations. Bhatwadekar et al. [28] similarly reported equivalent survival outcomes and therapeutic responses with generics. Dalle et al. [29] validated these findings in the USA, reinforcing the global applicability of generic imatinib. Cumulatively, these studies suggest that generic TKIs offer non-inferior efficacy and safety compared to branded counterparts, corroborated by our study.

Adverse effect profiling revealed neutropenia (8.2%) and anemia (5.9%) as the most common hematological toxicities. Leukopenia and thrombocytopenia were reported in 11% of patients along with anemia in 5% by Nair et al. [24]. Phukan et al. [17] observed higher rates of anemia (89.5%), neutropenia (75%), and thrombocytopenia (78.9%), potentially reflecting population or management differences. Non-hematological adverse effects predominantly included edema (14.1%) and fatigue (11.7%). Phukan et al. [17] reported edema in 62% and nausea/vomiting in 57% of patients, whereas Nair et al. [24] noted hyperpigmentation in 9% and pedal edema in 3%.

Mutation analysis of patients failing to achieve MMR revealed that among 32 non-responders, 15 (46.87%) tested positive for *KD* mutations, 10 (31.25%) were negative, and seven did not undergo testing due to financial constraints. Poor compliance was identified in five out of these seven untested patients. Of the *KD* mutation-positive cases, one (6.66%) was high-risk and 14 (93.33%) were intermediate-risk. Comparable mutation rates were reported by Majumdar et al. [30] (29.8%) and Gadhia et al. [31] (20.93%). *F317L* mutations were the most prevalent kind of mutation (53.33%), while combination mutations (*F317L* and *T315I*; *c.C944T* and *T315I*) were discovered in 13.33% of individuals. Gadhia et al. [31] discovered *T315I, M244V*, and *G250E* mutations, but S et al. [32] found *T315I* (24.24%), *Y253H *(15%), and *F317L* (12.12%) to be frequent mutations. The predominance of *F317L* in our cohort may reflect regional genetic variation, though the small sample size and use of Sanger sequencing could have under-detected low-allele variants.

Compliance assessment indicated that 10 (11.76%) patients were non-adherent to therapy, whereas 75 (88.23%) demonstrated good adherence. Cheng et al. [33] similarly found a 77.4% adherence rate. Noncompliance, influenced by socioeconomic barriers, likely contributed to suboptimal responses in a subset of patients. Compliance was objectively measured through pill count. Patients were educated about the course of disease, the importance of adherence to TKI, the possible side effects, and the necessity of regular monitoring of blood counts and molecular assessment.

Limitations

A significant drawback of our research is the limited number of participants in the single-center study and the relatively brief period of observation. One significant limitation of this study was the absence of in-house facilities to perform *BCR-ABL* transcript testing (qualitative and quantitative) and kinase domain mutation sequencing. Consequently, we were required to outsource these tasks to a partnered corporate laboratory, which routinely provides reports on *BCR-ABL1* transcript levels as IS normalized copy numbers (expressed as %) and *KD* mutations via Sanger sequencing.

## Conclusions

The current study, utilizing Indian generic TKIs, showcased significant clinical, hematological, and molecular responses. The drugs were found to be easily tolerated, and any negative effects observed were effectively managed with appropriate care. The Indian generic version is offered at a considerably reduced price, rendering it a more economical choice for CML-CP patients. The study's findings have the potential to greatly impact CML treatment strategies, shedding light on the advantages of using generic TKIs in settings with limited resources. The present study also highlighted the prevalence of the *KD* mutation in northeastern India.

The present study concluded that generic TKIs, primarily imatinib, offer therapeutic efficacy and safety profiles comparable to original formulations in the management of CML-CP. The use of ELTS scoring for risk stratification enhanced treatment individualization. While adverse effects were within expected ranges, non-compliance and *KD* mutations remain critical challenges affecting outcomes. This study was conducted over a period of one year and evaluated molecular responses at three, six, and 12 months intervals. Research is still ongoing to assess deep molecular responses, and those findings will be reported at a later stage. Continued monitoring, patient education, and broader access to mutation testing may further optimize therapeutic success in this patient population.
